# p31-43 Gliadin Peptide Forms Oligomers and Induces NLRP3 Inflammasome/Caspase 1- Dependent Mucosal Damage in Small Intestine

**DOI:** 10.3389/fimmu.2019.00031

**Published:** 2019-01-30

**Authors:** María Florencia Gómez Castro, Emanuel Miculán, María Georgina Herrera, Carolina Ruera, Federico Perez, Eduardo Daniel Prieto, Exequiel Barrera, Sergio Pantano, Paula Carasi, Fernando Gabriel Chirdo

**Affiliations:** ^1^Instituto de Estudios Inmunológicos y Fisiopatológicos (CONICET), Universidad Nacional de La Plata, La Plata, Argentina; ^2^Instituto de Fisicoquímica y Químicas Biológicas, Dr. Alejandro Paladini (CONICET), Universidad de Buenos Aires, Buenos Aires, Argentina; ^3^Laboratorio de Nanoscopía y Fisicoquímica de Superficies (CONICET), Universidad Nacional de La Plata, La Plata, Argentina; ^4^Biomolecular Simulations Group, Institut Pasteur de Montevideo, Montevideo, Uruguay

**Keywords:** enteropathy, celiac disease, inflammasome, caspase-1, p31-43, gliadin peptides, innate immunity, small intestine damage

## Abstract

Celiac disease (CD) is a chronic enteropathy elicited by a Th1 response to gluten peptides in the small intestine of genetically susceptible individuals. However, it remains unclear what drives the induction of inflammatory responses of this kind against harmless antigens in food. In a recent work, we have shown that the p31-43 peptide (p31-43) from α-gliadin can induce an innate immune response in the intestine and that this may initiate pathological adaptive immunity. The receptors and mechanisms responsible for the induction of innate immunity by p31-43 are unknown and here we present evidence that this may reflect conformational changes in the peptide that allow it to activate the NLRP3 inflammasome. Administration of p31-43, but not scrambled or inverted peptides, to normal mice induced enteropathy in the proximal small intestine, associated with increased production of type I interferon and mature IL-1β. P31-43 showed a sequence-specific spontaneous ability to form structured oligomers and aggregates *in vitro* and induced activation of the ASC speck complex. In parallel, the enteropathy induced by p31-43 *in vivo* did not occur in the absence of NLRP3 or caspase 1 and was inhibited by administration of the caspase 1 inhibitor Ac-YVAD-cmk. Collectively, these findings show that p31-43 gliadin has an intrinsic propensity to form oligomers which trigger the NLRP3 inflammasome and that this pathway is required for intestinal inflammation and pathology when p31-43 is administered orally to mice. This innate activation of the inflammasome may have important implications in the initial stages of CD pathogenesis.

## Introduction

Celiac disease (CD) is the commonest form of immune mediated enteropathy throughout the world and is caused by an antigen-specific CD4^+^ T cell response to wheat gluten in genetically susceptible individuals (1). Although a gluten-specific Th1 response is essential for the disease, there is increasing evidence that an initial innate immune response is necessary to trigger the local inflammation that drives adaptive immunity (2). As gluten proteins are not fully digested by gastrointestinal enzymes, long gluten-derived peptides remain in the gut lumen. Among these peptides, the α-gliadin 56–88 peptide (commonly named 33mer), is the model peptide for studying the adaptive response in CD (3). One candidate for driving this innate immune response is the gluten-derived peptide, α-gliadin 31-43 (p31-43) (4) which has been shown to induce several proinflammatory effects *in vivo* both in humans (5), and mice (6). Although p31-43 is thought to act by triggering an intracellular stress response because it interferes with endosomal trafficking (5), no receptor has been identified for this peptide (7) and therefore it is unknown if and how it can be recognized by the sensors of the innate immune system.

To unveil how this peptide induces inflammation and tissue damage, we aimed to evaluate whether a particular conformation may be responsible for its biological effects.

The special aminoacidic sequence of gluten proteins (8) as well as of shorter peptides as 33mer, confers uncommon conformational features such as polyproline II conformation and the occurrence of oligomers (9, 10). In the case of p31-43, the structure has not been studied.

Oligomeric structures and fibrils are triggers of inflammation through activation of the NLRP3 inflammasome leading to production of active IL-1β and cell death by pyroptosis. Nanostructures may alter intracellular organelles (such as endoplasmic reticulum and mitochondria) and release danger signals which can be sensed by the NLRP3 inflammasome (11).

Here, we show that p31-43 gliadin has an intrinsic propensity to form oligomeric structures that allow it to trigger the NLRP3 inflammasome, leading to caspase 1 dependent intestinal inflammation and pathology when administered orally to mice.

## Materials and Methods

### Mice

Male C57BL/6 were purchased from the Animal Care facility of the Veterinary Faculty from National University of La Plata, while caspase1/11^−/−^ (B6N.12952-*Casp1*^tmflv^/J) and NLRP3^−/−^ (B6.12956-*Nlrp3*^tm1Bhk^/J) mice were obtained from the Transgenic and Experimental Animal Unit of the Institut Pasteur de Montevideo (Uruguay). All mice were housed under specified pathogen free conditions and allowed access to autoclaved food and water *ad libitum*. Mice were used at 7 weeks of age and all experimental procedures were performed under appropriate local ethical guidelines. Experimental protocols were approved by the Institutional Animal Care and Use Committee of the Facultad de Ciencias Exactas, Universidad Nacional La Plata (Protocols 002-05-15 and 009-27-17) or by the Animal Ethics Committee of the Institut Pasteur (Montevideo) in accordance with national law 18.611 and international animal care guidelines regarding laboratory animal protocols (protocol 011-17).

### Peptides

The following peptides were synthesized at >95% purity by GeneCust, Luxembourg: p31-43 (LGQQQPFPPQQPY), non-related peptide (NRP, LDPLIRGLLARPACALQV), scrambled sequence peptide (SP, YQPLFQPQGPQPQ), and inverted sequence peptide (IP, YPQQPPFPQQQGL).

### Induction of Enteropathy by Administration of Peptides

Peptides were administered in a volume of 100 μl by intraluminal injection into the small intestine 2 cm from the pylorus after laparotomy under isofluorane anesthesia as described previously (6). Control mice received phosphate saline buffer (PBS). After surgery, fluid replacement was administered and mice were monitored until recovery. For oral administration, peptides, or PBS were given in 200 μl using a curved oral gavage needle (22G, 3.8 cm).

Four or 16 h post-inoculation, mice were euthanized, and intestinal samples were collected for mRNA evaluation and histological analysis. To inhibit caspase-1 activity *in vivo*, mice received 8 mg/kg Ac-YVAD-cmk (InvivoGen, United States) in DMSO (5% v/v) or DMSO alone intraperitoneally 30 min before oral administration of peptide. After 16 h, mice were euthanized and intestinal samples were collected for histological analysis.

### Histological Evaluation

Sections of proximal small intestine of mice were fixed in 4% w/v formalin, embedded in paraffin, and stained with H&E for histological evaluation using a Nikon Eclipse Ti fluorescence microscope with X-Cites Series 120 Q light source. Images were taken with a Nikon Digital Sight DS Ri1 camera using Nis-Elements software and measurements were performed using Image J software. Sections were scored in a blinded fashion, with at least 30 villus:crypt ratios assessed in each mouse, while intraepithelial lymphocytes (IELs) were counted in 10 randomly chosen villus tips and expressed as IELs/100 enterocytes.

### Real Time PCR

Small Intestinal samples from mice were stored in RNA Later (Ambion, United States) at −20°C until use and RNA was extracted using Illustra RNA spin mini RNA isolation Kit (GE Heathcare). cDNA was synthesized from 2 to 5 μg RNA using M-MLV Reverse Transcriptase (Invitrogen) and Real Time PCR was performed using SYBER green PCR Master mix (BioRad) for 40 cycles, with the following primers: HPRT-f 5′-CAATGCAAACTTTGCTTTCC-3′; HPRT-rev 5′- CAAATCCAACAAAGTCTGGC- 3′; IFNβ-f 5′-AATGGAAAGATCAACCTCAC-3′; IFNβ-rev 5′-AAGGCAGTGTAACTCTTCTG-3′. HPRT was used as an internal control to normalize the expression levels. All results were expressed as fold increase of each treatment vs. the mean of PBS treated samples for each time point using the 2^−ΔΔ*Ct*^ method). All samples were assessed in triplicate.

### Activation of the Inflammasome *in vitro*

ASC reporter cell line (InvivoGen) were kindly provided by Dr. Rumbo M (IIFP). Cells were cultured in RPMI 1640, 25 mM HEPES/2 mM L-glutamine/10% heat-inactivated fetal bovine serum/50 U/ml penicillin, 50 μg/mL streptomycin at 37°C in 5% CO_2_. 180 μl aliquots of 2 × 10^6^ cells/mL were added to a 96-well plate and allowed to adhere for 1 h, before 20 μl of LPS at 1 μg/mL was added for 3 h to “prime” the inflammasome. Medium was then removed and replaced by fresh medium containing 50 or 100 μg/mL p31-43 or inverted peptide, before the cells were cultured for 16 h at 37°C. After stimulation, the cells were washed with PBS and fixed with 2% PFA for 15 min at 37°C and stained with 1 μg/mL DAPI to visualize the nucleus. ASC specks were then imaged by assessing GFP expression using a Nikon Eclipse Ti fluorescence microscope and Nikon Digital Sight DS Ri1 camera with Nis-Elements. Results were expressed as ASC positive cells/100 cells. All treatments were performed in triplicate.

### Western Blot

Fresh small intestine tissues from individual mice were lysed and protein extracts were quantified using the BCA kit (Thermo, Rockford, United States). Protein samples (30 μg/lane) were separated by sodium dodecyl sulfate polyacrilamide gel electrophoresis on 10 or 12.5% gels and transferred onto nitrocellulose membranes. The membranes were blocked with 5% fat-free dry milk in TBST 0.1% at 37°C for 1 h and incubated with the following primary antibodies: anti-caspase 1 (1/150, SC-56036, Santa Cruz), anti-IL-1β (1/100, P420B, Invitrogen), anti-β-actin (1/2000, Ab8227, Abcam) at 4°C overnight. After washing, membranes were incubated with horseradish peroxidase (HRP)-conjugated secondary goat anti-rabbit antibody (1/2000, 1706515, BioRad), or horseradish peroxidase (HRP)-conjugated secondary anti-mouse antibody (1/3000, Ab6789, Abcam) at 37°C for 1 h, and visualized using the enhanced chemiluminescent reagent (GE Healthcare). The relative levels of the target protein to the control β-actin were determined using ImageJ software.

### Circular Dichroism

Circular Dichroism spectra of peptide solutions were recorded on a Jasco J-810 spectrometer polarimeter using a Peltier system as temperature controller. Five scans were acquired in the range of 190–250 nm at 4°C, using a scanning speed of 50 nm/min. The spectra were obtained employing 1 and 0.1 mm quartz cuvettes. Blank scans were subtracted from the spectra and smooth noise reduction was applied when needed using a binomial method (12). The data were expressed as the mean residue molar ellipticity in deg cm^2^ dmol^−1^. Graphics were represented using Origin Software (OriginLab).

### Transmission Electron Microscopy

Three aliquots of 50 μM and three of 100 uM concentration of p31-43 were prepared in MilliQ water from a 3 mM mother solution of the peptide. These solutions were deposited onto copper grids (200 mesh) coated with Formvar. After 5 min the excess fluid was removed by capillarity and the samples were negatively stained with 2% uranyl acetate in water for 2 min. After removal of excess fluid, the samples were allowed to dry and visualized using a JEOL 100CX II microscope operating at 100 kV.

### Modeling of Peptide Conformation *in silico*

Molecular dynamics simulations were performed at a coarse grained level employing the SIRAH force field (13). The initial structure of p31-43 was arbitrarily assumed to be a canonical α-helical conformation, with the N- and C-terminal amino acids considered as zwitteronic. An isolated peptide was simulated for 500 ns, setting a salt concentration (NaCl) to 150 mM. Four different structures were randomly chosen within this time window to serve as starting conformers and 50 copies of these conformers were disposed in a computational solvation box at a minimum distance of 4 nm between their centers of mass and simulated for 5 μs. Both the monomer and 50-mer simulations were performed with GROMACS 4.6.7 in the NPT ensemble at 310 K. The last 100 ns of each simulation were used for analysis and secondary structure was calculated using SIRAH tools (14) while interpeptide contacts were computed with g_mindist included in the GROMACS analysis tools. The aggregation was monitored as a function of the formation and sizes of clusters. A cluster was defined when the distance between two atoms of different peptides was <0.4 nm and mean cluster size (MCS) was calculated as MCS = (∑Ni = 1 CSi,t) /N, where CSi, t is the cluster size to which peptide i belongs at time t, and N is the total number of peptides (14).

### Statistical Analysis

Statistical analysis was performed with Graph-Pad Prism software (San Diego, United States). When two groups were compared, an unpaired Student's *t*-test was used, while a one-way ANOVA, post-test Bonferroni was used when more than two groups were compared. *P* <0.05 was considered significant. Data are displayed as means ± 1 SEM.

## Results

### Intraluminally Delivered p31-43 Gliadin Peptide Induces Mucosal Damage in a Sequence Specific Manner

**Figure 1:**
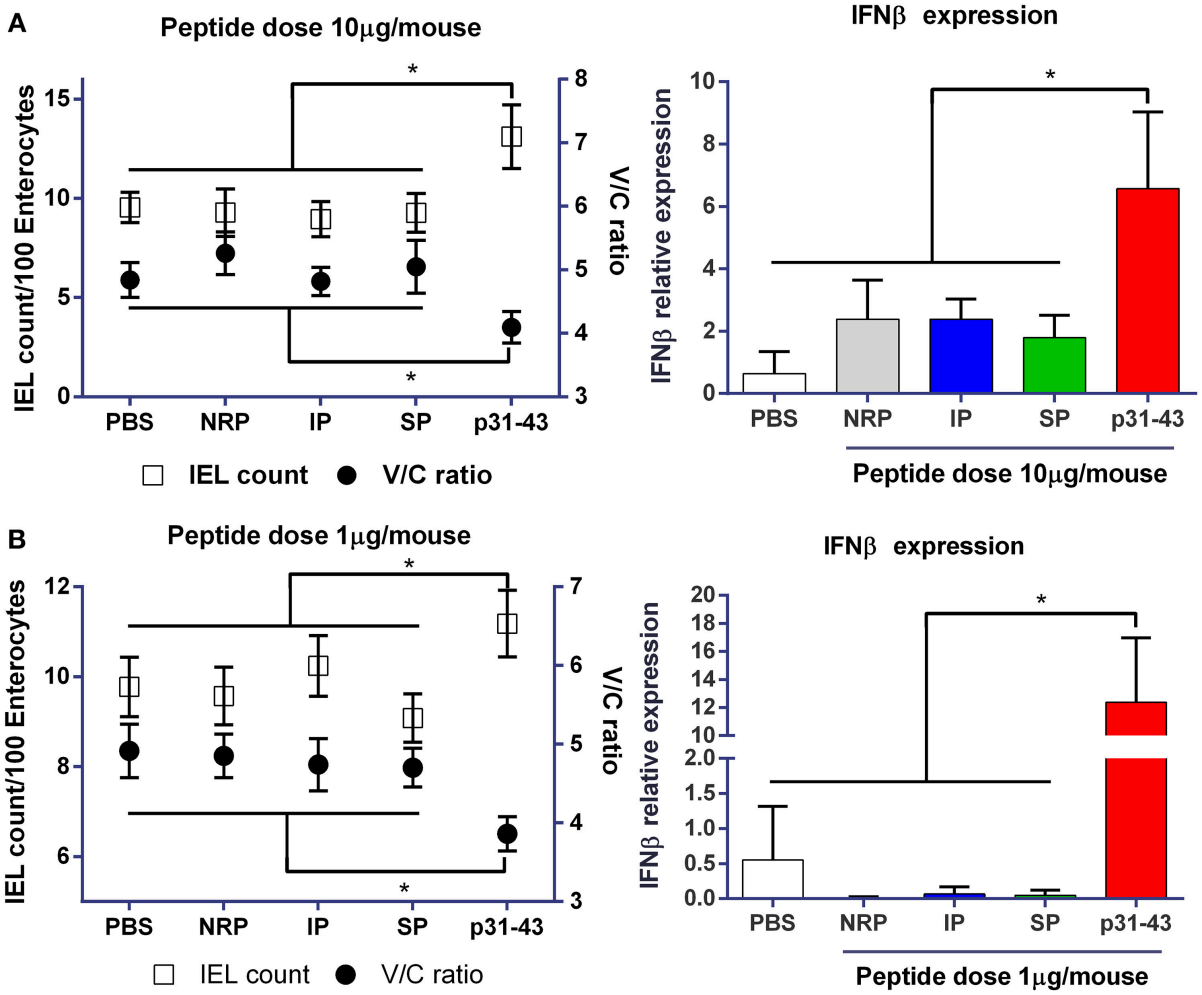
Gliadin peptide p31-43 induces small intestine pathology in a sequence-specific manner. C57BL/6 mice were injected intraluminally with native p31-43, inverted sequence peptide (IP), scrambled sequence peptide (SP), non-related peptide (NRP), or PBS. The ratio between villus length and crypt depth (V/C) (right axis) and the numbers of intraepithelial lymphocytes/100 enterocytes (left axis) were determined on H&E stained sections of proximal small intestine 16 h after administration of 10 μg **(A)** or 1 μg **(B)** peptide/mouse. IFNβ mRNA was assessed by RT-qPCR on whole proximal small intestine samples 4 h after treatment. Results shown are means ± 1 SEM for 5 mice per group. ^*^p < 0.05 by ANOVA, post-test Bonferroni.

To explore how the sequence and structure of p31-43 might influence its ability to induce enteropathy, we first compared the effects of intraluminal administration of the native peptide (LGQQQPFPPQQPY) and synthetic peptides with an inverted (YPQQPFPPQQQGL) (IP) or scrambled sequence (YQPLFQPQGPQPQ) (SP). This route was used to avoid potentially complicating effects of the peptides being differentially sensitive to cleavage by gastrointestinal enzymes. An unrelated peptide (NRP) and PBS (vehicle) were also used as controls. As we have shown previously (6), 16 h after intraluminal administration of 10 μg native p31-43, mice showed evidence of pathology in the upper small intestine, with a lower villus length/crypt depth (V/C) ratio and a higher number of intraepithelial lymphocytes (IELs) compared with PBS controls (Figures 1A,B). p31-43 treated mice also showed increased expression of mRNA for IFNβ in the mucosa 4 h after treatment (Figure 1A). Similar findings were observed when mice were treated with 10 times less amount of native p31-43 (Figure 1B), suggesting that this peptide has a potent biological *in vivo* effect on the small intestinal mucosa. However, none of these intestinal changes were observed after administration of 10 μg of the inverted or scrambled peptides, indicating that the effects of p31-43 are sequence-specific and not a consequence of inflammation due to the surgical procedure.

### p31-43 Peptide Adopts a Polyproline II Structure and Self-Organizes in Oligomers

**Figure 2:**
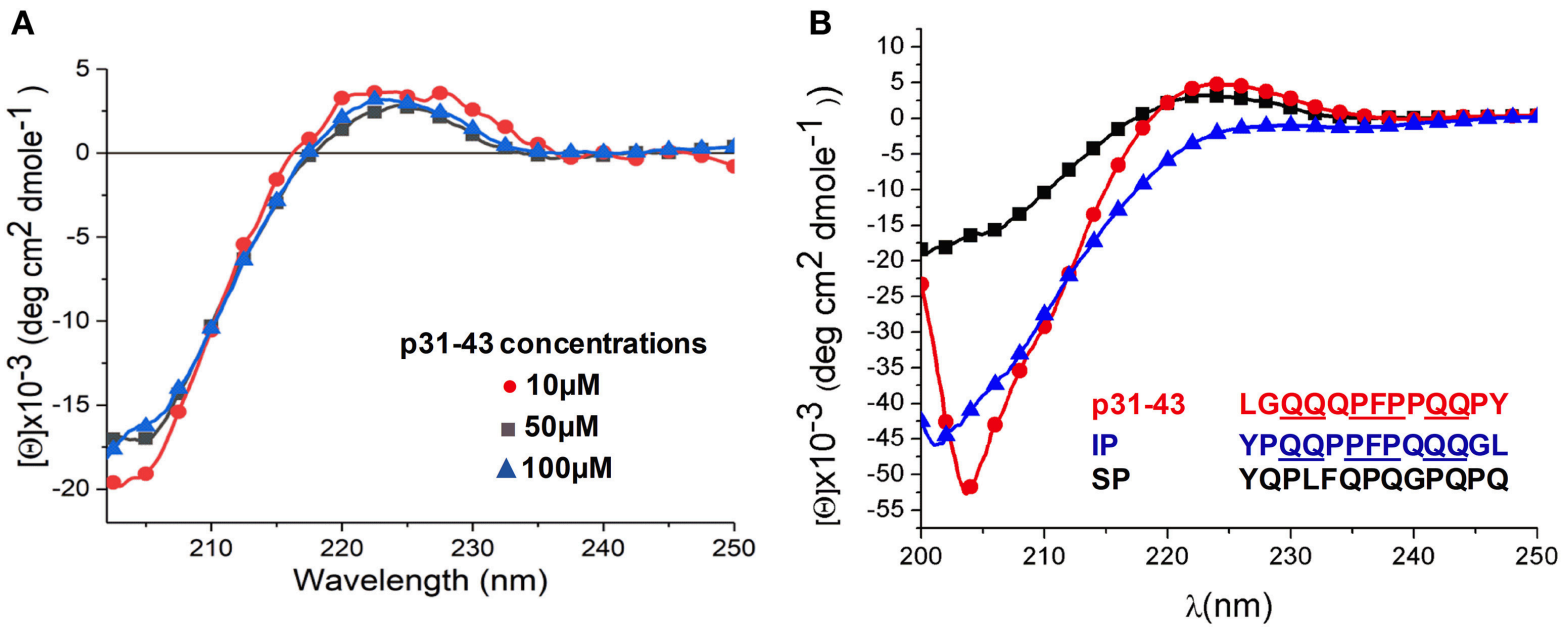
Structural analysis of p31-43 by circular dichroism shows polyproline II structures. **(A)** Circular dichroism spectra obtained using 10 μM (15.3 μg/mL), 50 μM (76.5 μg/mL), and 100 μM (153 μg/mL) solutions of peptide at 4°C. A negative band at 205 nm together with a positive one 225 nm indicates the presence a polyproline II structure, while the hypochromic shift of the bands indicates a self-assembly process. Results shown are representative of three independent experiments. **(B)** Circular dichroism analysis of p31-43, inverted peptides (PI) and scrambled peptides (SP) at 100 μM (153 μg/mL) at 4°C. In the scrambled peptide a negative band at 203 nm is indicative of a random secondary structure, while the negative band at around 204 nm and the positive one at 225 nm in the p31-43 and inverted peptides is indicative of a polyproline II conformation. p31-43 shows a hypochromic displacement of the negative band with respect to the inverted peptide, suggesting a self-organization process in p31-43. Results are representative of three independent experiments.

Together with our previous findings that p31-43 activates the MyD88 and the type I IFN signaling pathways (6), the results above indicate that p31-43 is a potent trigger of the innate immune response and that its sequence is critical for this effect. For this reason, we hypothesized that p31-43 may adopt a particular secondary structure that is responsible for its biological effects. To assess the secondary conformation of p31-43, Circular Dichroism analysis was performed on 10, 50, and 100 μM solutions of the peptide at 4°C, concentrations that are in the range used for the *in vivo* studies (Figure 2A). Under these conditions, p31-43 generated a signal characteristic of a polyproline II structure, as shown by a negative band near 203 nm and a positive band at 225 nm (15). When the concentration of p31-43 was increased, a small hypochromic displacement of the negative and the positive bands was detected, indicating a concentration dependent, self-organization process (16). When the scrambled and inverted peptides were analyzed at 100 μM concentration for comparison, the scrambled peptide showed only a negative band at 200 nm, indicative of a random coil structure. Although the inverted peptide showed evidence of a polyproline II-like structure, it did not show the hypochromic displacement seen with the native p31-43 peptide (Figure 2B).

**Figure 3:**
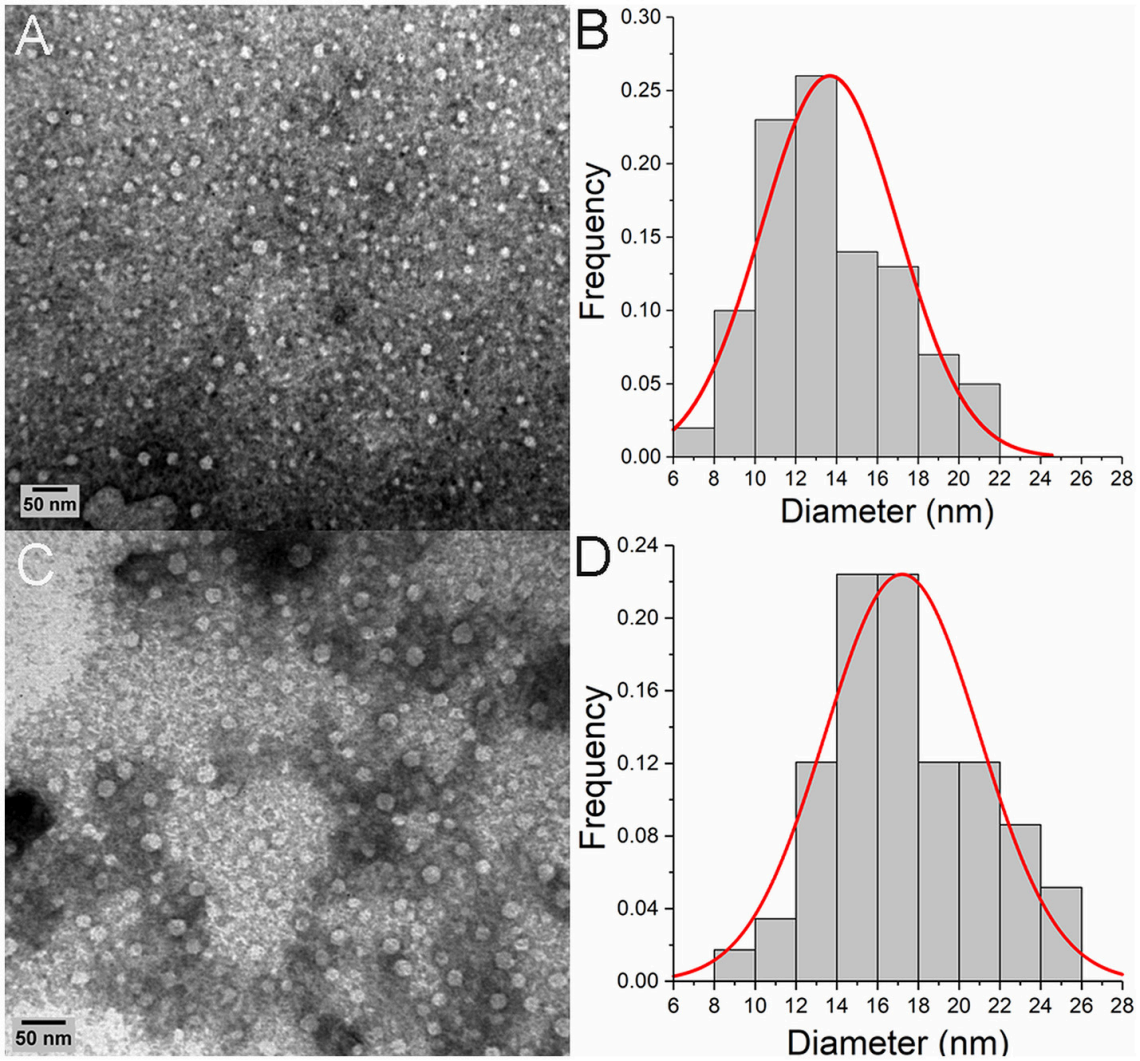
Oligomeric p31-43 structures characterized by TEM. Samples of p31-43 were prepared in MilliQ water and deposited on a grid covered with formvar. **(A)** Representative image showing the presence of oligomeric structures in a 50 μM (76.5 μg/mL) solution. **(B)** Size distribution of the oligomers observed in **(A)**, showing a mean diameter of 13.6 ± 3.4 nm. **(C)** Representative image showing the presence of oligomers in a 100 μM (153 μg/mL) solution. **(D)** Size distribution of the oligomers observed in **(C)**, showing a mean diameter of the structures is 17 ± 7 nm.

Given this evidence that p31-43 might show a selective ability to self-organize in solution, we next used transmission electron microscopy (TEM) to analyze this in more detail. This showed that at 50 μM concentration, the peptide formed spherical oligomers randomly distributed (Figure 3A). These oligomers had an average diameter of 13.6 ± 3.4 nm (Figure 3B), while the similar oligomers formed at 100 μM had higher mean diameters (17 ± 7 nm) (Figures 3C,D).

Together these studies show that p31-43 peptide can adopt a polyproline II structure which is able to self-organize and generate complex nanostructures *in vitro*.

### *In silico* Modeling of p31-43 Oligomers

**Figure 4:**
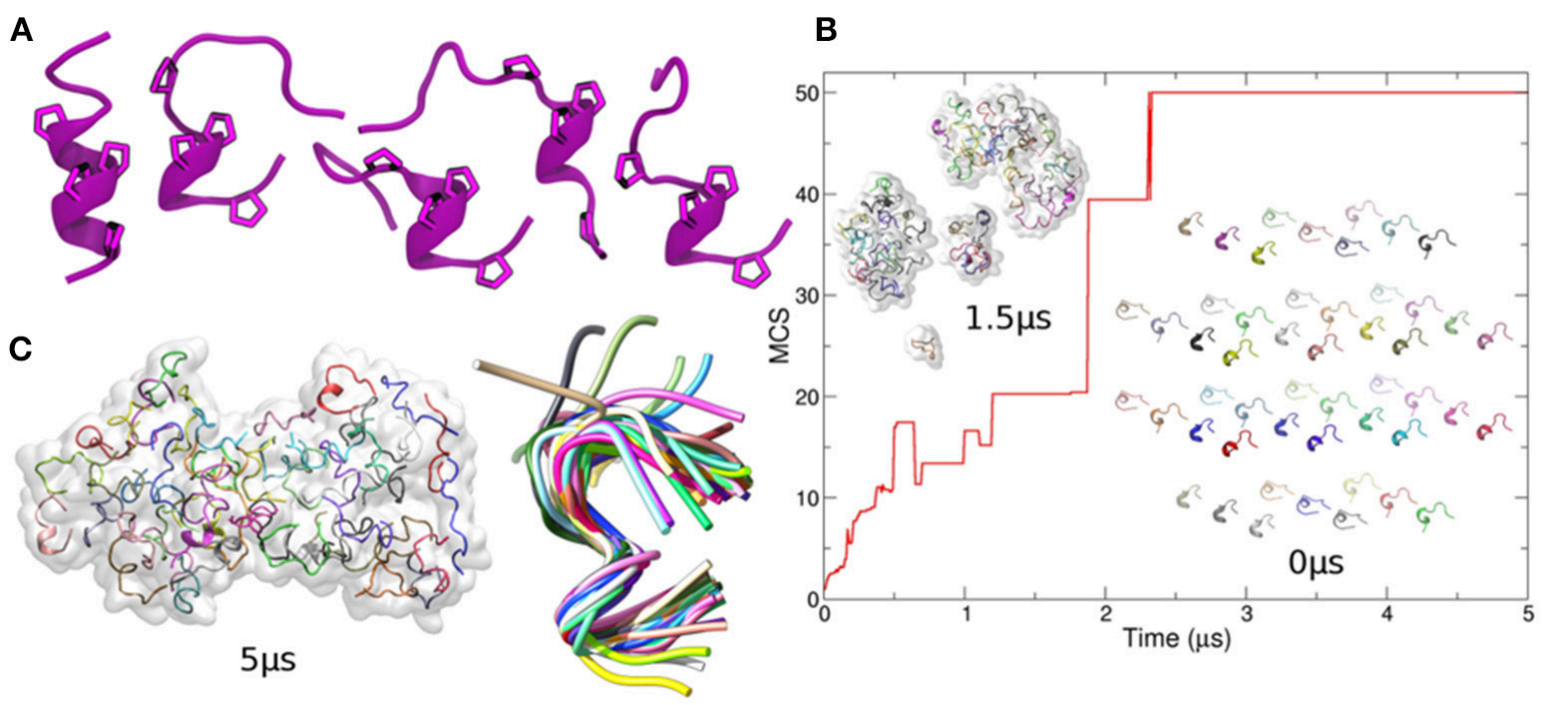
*In-silico* modeling of p31-43 aggregation. **(A)** Left: cartoon representation of the initial (helical) conformation used as starting conformer. For visual clarity, only Prolines are shown. Right: four different conformers arbitrarily chosen along the simulation of the isolated peptide and used to generate the initial configuration of the system containing 50 copies of p31-43 [inset indicated with 0 ms in panel **(B)**]. The cartoons show the conserved presence of polyproline. **(B)** Mean cluster sizes of aggregates formed before (bottom right) and during simulation (top left) using 50 copies of p31-43. Individual peptide copies are identified by different colors. **(C)** The final state is shown (left), with structural convergence within the aggregate being highlighted by superposition of individual copies within a maximum root mean square deviation of 0.1 nm, which corresponds to 41 out 50 peptides (right).

To gain further insights into the molecular determinants of p31-43 aggregates, we undertook *in silico* modeling of the structures formed by the peptide. When a single copy of the peptide was arbitrarily assumed to start in a helical state, it was found to be highly flexible (Supplementary Movie 1), spontaneously acquiring random coil conformations, with the exception of the central Pro36, Phe37, Pro38 residues, which retained a nearly helical conformation (Figure 4A). In contrast, when multiple copies of p31-43 were simulated together, they underwent spontaneous aggregation, via formation of small clusters that eventually fused into one single aggregate (Figure 4B). The non-monotonic variation of the mean cluster size suggests that spontaneous dissociation events also take place in solution. The final aggregates had a prolated shape, with major and minor diameters of 10.1 nm ± 0.2 and 6.9 nm ± 0.1, respectively, comparable to the smaller oligomers found by TEM (Figure 3). In contrast with the isolated peptide, multimer p31-43 copies showed structural convergence, as shown by superposition of individual monomers in the final conformation (Figure 4C and Supplementary Movie 2). These aggregates exhibited an increase in the amount of polyproline II conformation compared with the monomers. Glutamine-glutamine interactions accounted for around one fifth of the total contacts between individual peptide strands in the aggregate (20% ± 1.6), while glutamine residues were not involved in almost half of these contacts (45% ± 1.8). This suggests that although the peptide may have an intrinsic tendency to form aggregates, these may have a rather loose structure.

### p31-43 Activates the Apoptosis-Associated Speck-Like (ASC) Complex *in vitro* and Drives Production of IL-1β *in vivo*

We next asked whether activation of the innate immune response and induction of enteropathy by p31-43 were related to its ability to form aggregates with a defined nanostructure. The nucleotide-binding domain, leucine-rich repeat-containing-like receptors (NLRs) are cytosolic sensors that drive innate immunity by mediating recognition of other kinds of particulate materials including protein fibrils and of signals derived from organelles damaged by these material (11). Therefore, we examined the role of the NLRP3 inflammasome and its downstream effector molecule caspase-1 in the effects of p31-43.

As we wished to avoid potentially confounding effects of surgery on the activation of the NLRP3 inflammasome, we decided to use the oral route for administering peptide in these studies. Preliminary experiments showed that administration of 2–20 μg p31-43 to wild type C57Bl/6 mice by oral gavage reproduced the changes in V/C ratio and number of IELs in the proximal small intestine that we had found when p31-43 was given intraluminally (Figure 7 and Supplementary Figure 1). The effects were dose dependent and we used the optimal dose of 20 μg peptide in subsequent experiments.

**Figure 5:**
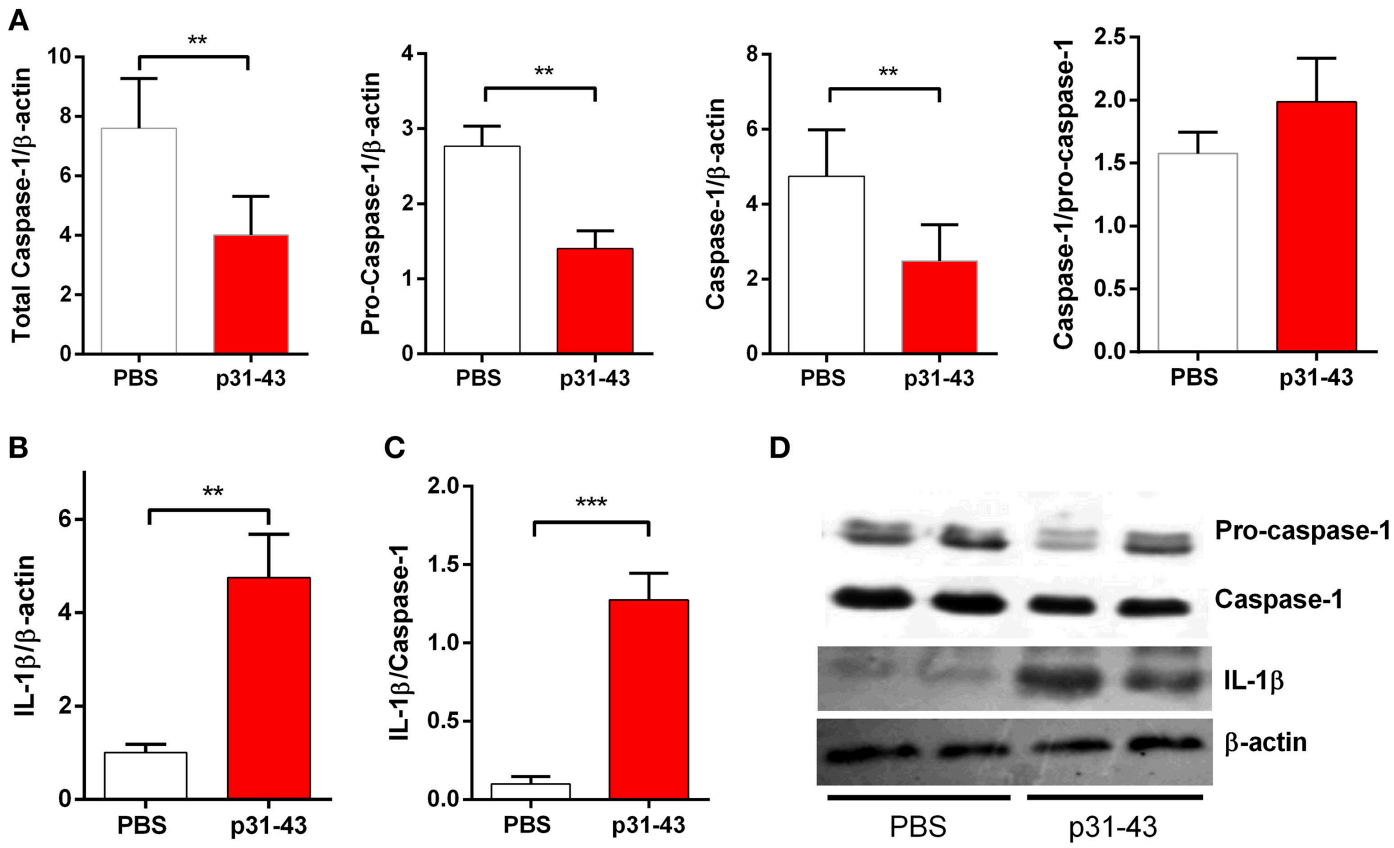
Oral administration of p31-43 induces caspase 1 activation and production of mature IL-1β. **(A)** Western blot analysis of mature IL-1β and **(B)** caspase-1 and pro-caspase-1 quantification in whole small intestinal tissue from p31-43 or vehicle-fed mice 16 h after treatment. **(C)** IL-1β/caspase 1 ratio of data shown in **(A,B)**. **(D)** Representative results of Western blots are shown. Results shown are means ± 1 SEM for 5 mice and are representative of two experiments. ^**^p < 0.01, ^***^p < 0.001, Student's *t*-test.

The changes in V/C ratio and IEL counts found 16 h after oral administration of 20 μg p31-43 to WT mice were accompanied by increased mucosal levels of mature IL-1β, together with a parallel decrease in the levels of pro-caspase 1, and correlation between caspase 1 and IL-1β levels (Figure 5). Thus, the increment of IL-1β indicates the activation of the inflammasome caspase-1 pathway in the small intestine mucosa by oral administration of p31-43.

**Figure 6:**
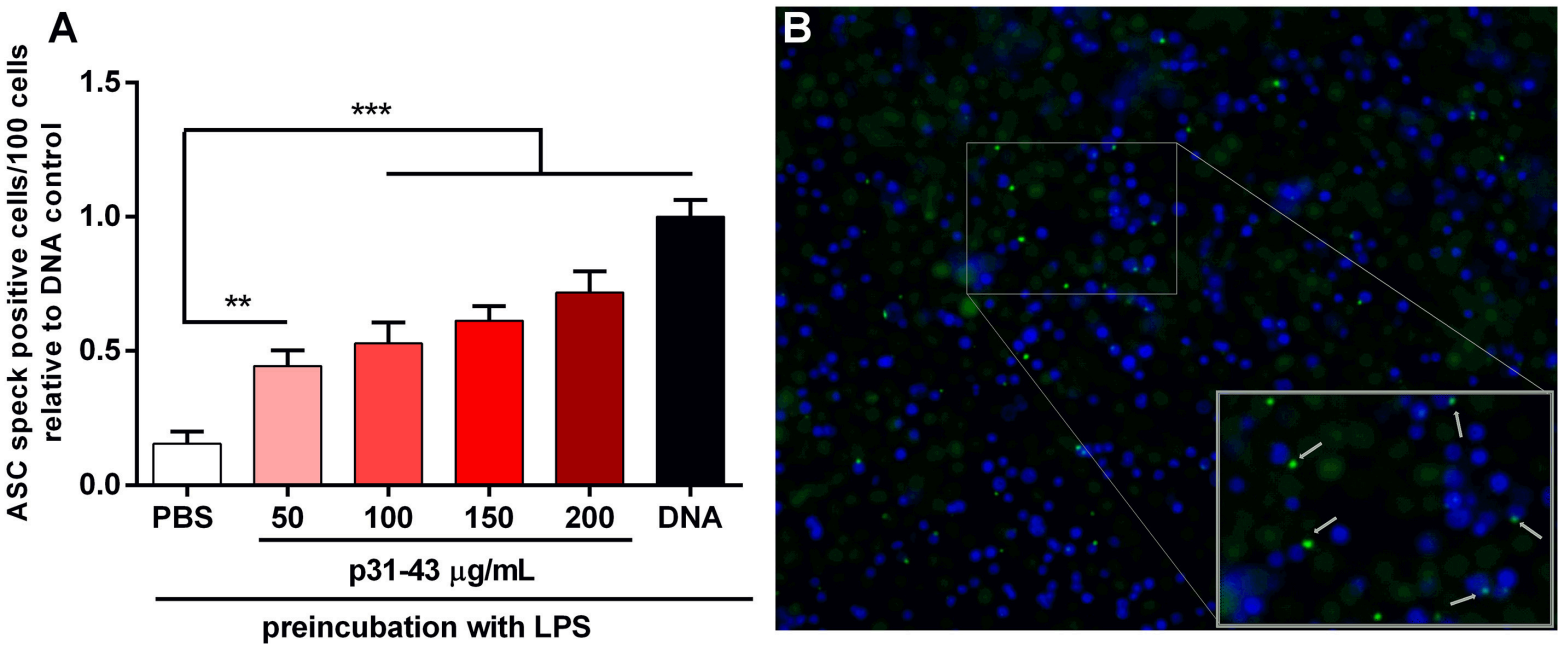
p31-43 induces ASC speck formation *in vitro*. **(A)** Human THP-1 ASC speck reporter cells were preconditioned for 3 h with LPS before addition of different amounts of p31-43 for a further 16 h. Positive control cells (DNA) were transfected transiently with bacterial plasmid before addition of LPS. Cells showing ASC specks were assessed by immunofluorescence and expressed as positive cells/100 total cells. Results shown are means ±1 SEM for three replicates/group and are representative of two experiments ^**^p < 0.01; ^***^p < 0.0001, ANOVA. **(B)** Representative images of ASC speck expressing cells (green) (blue–DAPI).

The inflammasome acts by recruiting a protein complex that contains the apoptosis-associated speck-like (ASC) protein. Therefore, to confirm that p31-43 can activate this complex, we used a THP1 cell line expressing ASC-GFP that allowed the resulting specks to be visualized by fluorescence microscopy. In these experiments, the inflammasome was first “primed” by pre-incubation of the THP1-ASC-GFP cells with LPS before addition of peptide. Under these conditions, p31-43 induced dose dependent increases in speck formation compared with cells challenged with PBS alone (Figure 6). This effect was not observed with the inverted peptide (Supplementary Figure 2).

### Small Intestinal Damage Induced by p31-43 Requires NLRP3 and Caspase-1

**Figure 7:**
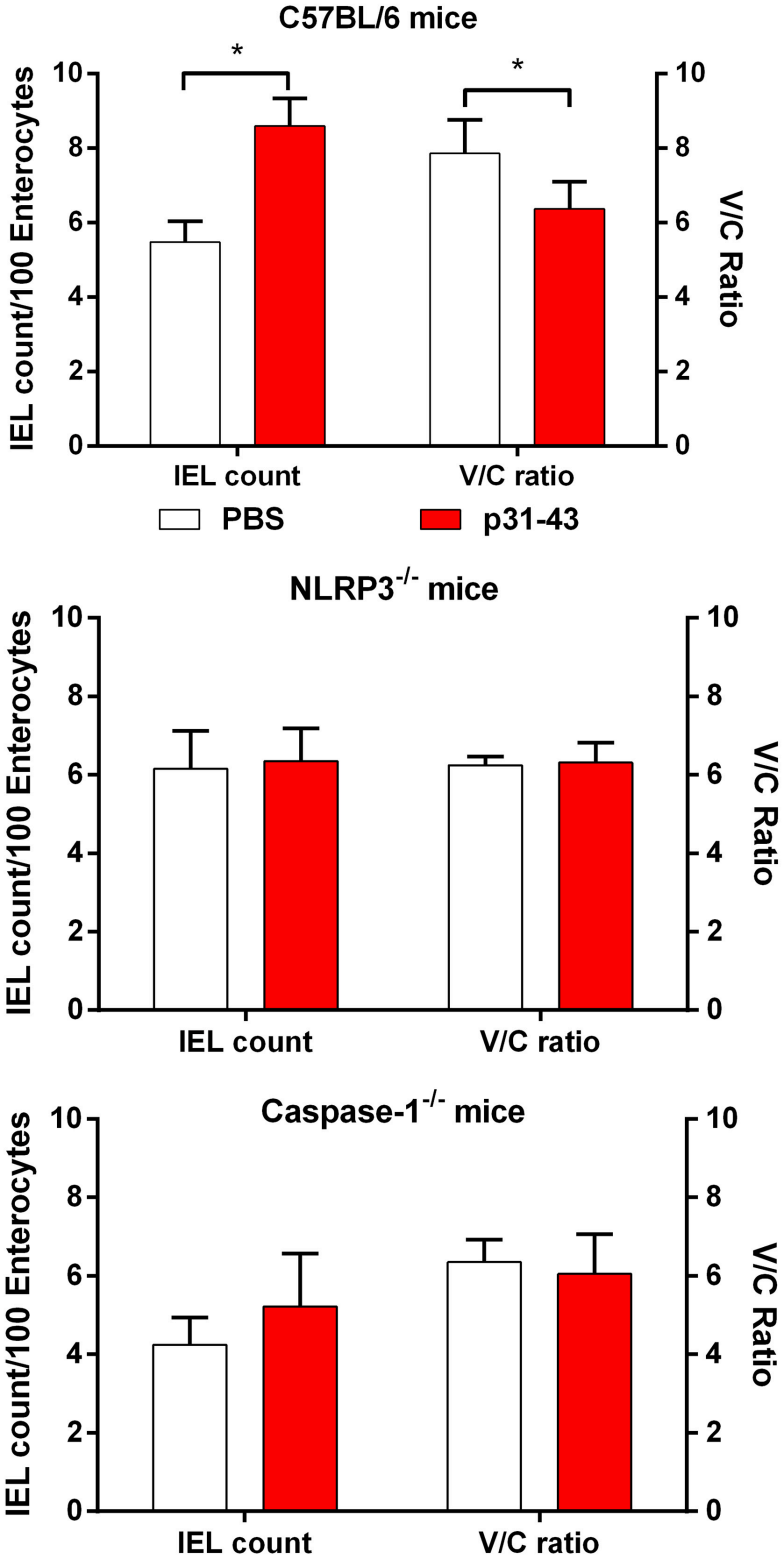
Enteropathy induced by p31-43 requires NLRP3 and caspase 1. Morphological analysis of proximal small intestine of C57BL/6, *NLRP3*^−/−^, and *caspase 1*^−/−^ mice, 16 h after oral administration of 20 μg p31-43 or PBS, showing mean IEL/100 enterocytes (left axis) and villus/crypt ratios (right axis). Results shown are means ± 1 SEM for 6 mice/group. ^*^p < 0.05.

**Figure 8:**
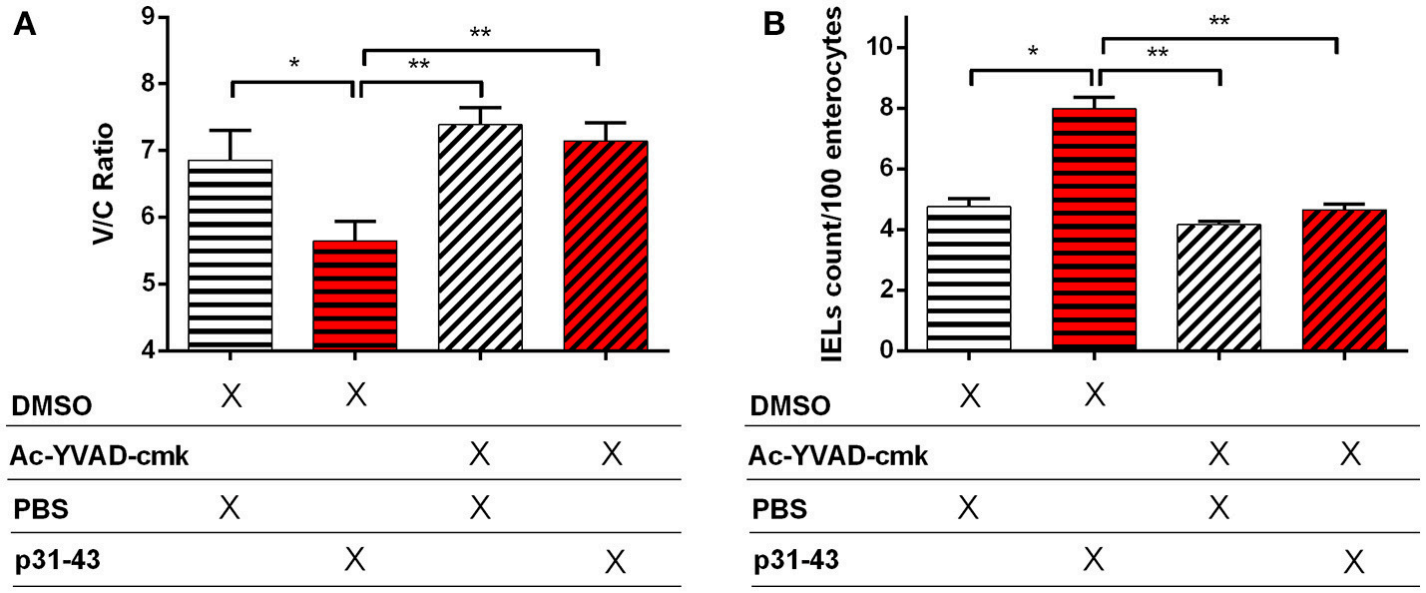
Enteropathy induced by p31-43 is prevented by inhibition of caspase 1. Villus/crypt rations **(A)** and intraepithelial lymphocyte counts **(B)** in the proximal small intestine of C57BL/6 mice fed 20 μg p31-43 or PBS that had been treated ip 30 min earlier with either 8 mg/kg of the Ac-YVAD-cmk caspase 1 inhibitor or with DMSO. Results shown are means ± 1 SEM for 5 mice/group assessed 16 h after feeding p31-43 and are representative of two experiments. ^*^p < 0.05, ^**^p < 0.01, ANOVA, Post-test Bonferroni.

As these results suggested that p31-43 might activate the inflammasome *in vivo* and *in vitro*, we assessed whether the NLRP3 inflammasome was involved in the pathological effects of p31-43 in the small intestine. In support of this idea, *Nlrp3*^−/−^ and *caspase-1*^−/−^ mice did not develop altered V/C ratios or increases in IELs 16 h after feeding p31-43 (Figure 7) and administration of the irreversible inhibitor of caspase-1, Ac-YVAD-cmk, prevented these changes in WT mice fed p31-43 (Figure 8 and Supplementary Figure 3). Thus, the enteropathy induced *in vivo* by p31-43 is dependent on NLRP3 and caspase 1.

## Discussion

Due to their unusual sequence, gluten peptides are resistant to proteolytic enzymes in the gastrointestinal tract and several peptides of reasonable length may remain undigested in the intestinal lumen. Amongst these is the p31-43 peptide from gliadin which has been detected intact after extensive digestion by a combination of gastric, pancreatic, and brush border enzymes (17). *In vitro* and *ex vivo* assays have shown that p31-43 gliadin has proinflammatory and toxic effects (5). De Ritis et al. (18) reported morphological alterations in enterocytes when duodenal samples from untreated CD patients were incubated with p31-55 gliadin peptide, which contains the p31-43 sequence. Using organ culture, it was observed that p31-43 induces the expression of HLA-DR molecules, a sign of inflammatory response, in epithelial cells in the crypts (19) and the expression of IL-15, CD83, cyclo-oxygenase 2 in *lamina propria* cells together with enterocyte apoptosis (4, 5).

Efforts to identify a receptor for p31-43 have been unsuccessful and recent reports suggest that it may be internalized into early endosomes after direct interaction with the cell membrane (7, 20) There, the peptide interferes with vesicle trafficking leading to overactivation of the IL-15 and EGF pathways (21) and also to ER-stress (22). We have shown previously that intestinal administration of p31-43 produces pathology in the small intestine, characterized by reduction in the V/C ratio, increased number of IELs, induction of inflammatory mediators and cell death in the proximal small intestine. Although all these effects were MyD88 and Type I IFNs dependent (6) it remains unclear how a 13 amino acid peptide can have such potent effects on the innate immune system.

As this gliadin-derived peptide consists of an unusual amino acid sequence, with a high content of glutamine and proline residues (8) we explored if this was related to its immunological properties. The assessment of conformational structure of gluten proteins by different techniques (12, 23, 24) showed that the large repetitive regions are in balance between β-turn and polyproline II conformations, while non-repetitive regions adopt α-helix or random conformation.

Our first experiments showed that the effects of p31-43 *in vivo* were not replicated by peptides with an inverted or scrambled sequence, suggesting that a specific conformation of this sequence might be the critical property. To test this idea, we used biophysical approaches to evaluate the structure of p31-43. Circular dichroism analysis showed that p31-43 presents a polyproline II secondary structure in all the concentrations we studied. These structural characteristics were not observed for the scrambled peptide, but were found with the inverted peptide which shares many critical positions of the p31-43 sequence (xxQQxPFPxQQxx). Therefore, these residues may drive the formation of a polyproline II structure. However, an important difference between the peptides was that only the native one showed hypochromic properties when the spectra were compared, suggesting that it may undergo self-assembly in solution. Similar behavior has been reported for a number of other proteins and peptides that are able to self-organize, such as the amyloidogenic exon-30 of tropoelastin (16) and the 33-mer gliadin peptide (9). These findings were supported by TEM analysis which showed that p31-43 spontaneously forms oligomeric nanostructures, whose size depends on the peptide concentration. The results also suggested that the larger aggregates may be generated by association or coalescence of smaller ones, a self-assembly process that has been found using similar approaches with proteins and peptides such as β-amyloid (25), elastin like peptides (26), and the 33-mer gliadin peptide (9).

Coarse grained molecular dynamic simulation *in silico* confirmed the tendency of p31-43 to adopt a polyproline II structure, before assembling spontaneously into small clusters and then into larger structures. Altogether, *in silico* studies correlating with results obtained by Circular Dichroism, Transmission Electron Microscopy and Atomic Force Microscopy (not shown) point out that p31-43 is able to self-organize and form nanostructures (Herrera et al., manuscript in preparation). Formation of a polyproline II structure appears to be a critical component of the pathology in diseases associated with accumulation of misformed proteins such as neurodegenerative diseases (27). Additional studies by CD, AFM, DLS and molecular simulations extended the characterization of p31-43 structure (Herrera et al., manuscript in preparation). This ability to form ordered nanostructures may help explain previous findings that p31-43 resists lysosomal degradation, preventing vesicular trafficking and maturation, leading to destabilization of vesicles, intracellular stress and increased levels of reactive oxygen species (20, 28). As there is no evidence for a specific receptor interacting with p31-43 (7) it was unclear how this process might provoke an innate immune response and one possibility we considered was activation of the NLRP3 inflammasome. This family of cytosolic pattern recognition receptor (PRR) complexes mediates the innate immune response to other particulate materials such as uric acid crystals and after intracellular stress insults such as mitochondrial damage or changes in local K^+^ levels (11). Furthermore, the NLRP3 inflammasome has been linked to chronic inflammatory disorders based on protein aggregation such as Alzheimer's and Parkinson's diseases (29). Here, we extend these findings by showing that p31-43 activated the inflammasome in THP1 macrophage-like cells *in vitro* and that the acute mucosal damage induced by p31-43 *in vivo* was absent in mice lacking NLRP3 or its downstream mediator caspase-1. Pharmacological inhibition of caspase-1 also prevented p31-43 induced pathology in wild type mice. In parallel, this acute mucosal injury was associated with increased levels of mature IL-1β, a cytokine whose production requires cleavage by caspase-1 and is a characteristic feature of inflammasome dependent innate immune responses (30). Interestingly, previous studies have reported that a complex mixture of gluten peptides stimulated IL-1β release by peripheral blood mononuclear cells from Celiac disease patients and that their analogous effects on murine bone marrow derived dendritic cells required NLRP3 (31, 32). Our findings indicate that the p31-43 peptide of α-gliadin may account for at least part of this effect, although whether other components of gluten also contribute remains to be determined.

In conclusion, we propose that p31-43 plays a central role in the pathogenesis of Celiac disease at least in part via an intrinsic ability to form oligomers of polyproline II-like structures that self-assemble and drive an NLRP3-caspase 1 dependent innate immune response in the intestinal mucosa. However, it is important to note that a variety of other triggers may be required to initiate overt inflammation in the mucosa, either alone or acting in synergy with each other. Indeed, it is known that activation of the inflammasome occurs in two stages, “priming” by PRR mediated activation of NF-κB activation, followed by the NLR ligands themselves (30). In this respect, we have shown previously that the MyD88 is required for the biological effects of p31-43 *in vivo* (6). The type I IFN pathway is a further aspect of the innate immune response that is induced by p31-43 *in vivo* (6) and high levels of expression of IFNα and the type 1 IFN pathway adaptor protein MxA are seen in duodenal mucosa from Celiac disease patients (33). Inflammatory signals triggered via the IFNαR, perhaps in enterocytes, could amplify further the innate immune response induced by p31-43. Further studies are needed to characterize the cells participating in the sensing of p31-43 nanostructures. It is also important to note that p31-43 may not be the only stimulus of innate immunity in Celiac disease, with other possibilities including wheat amylase trypsin inhibitors (34), viral infections (35), and bacterial dysbiosis (36). Generation of a local inflammatory response by factors such as these favors the induction of adaptive immune responses to gluten in genetically susceptible individuals, leading to permanent disease. It is tempting to speculate that p31-43 may also contribute to the pathogenesis of non-celiac gluten sensitivity (NCGS) another form of gluten intolerance with a high prevalence (up to 6% of general population) whose pathogenesis remains to be determined. However, it has been suggested that it may reflect an innate response to gluten occurring in isolation without generation of the adaptive immune response seen in CD (37). Together these results underline the importance of understanding the innate response and its triggers in different gluten-related disorders.

## Author Contributions

FC designed the study. MG, MH, SP, EP, PC, and FC wrote the manuscript. MG, EM, and PC performed the *in vivo* assays. MH, MG, and EP performed the biophysical studies. EB and SP performed the *in silico* studies. EM and FP performed ASC speck experiments. CR performed the western-blot analysis.

### Conflict of Interest Statement

The authors declare that the research was conducted in the absence of any commercial or financial relationships that could be construed as a potential conflict of interest.

## Abbreviations

NLRP3: nucleotide-binding oligomerization domain leucine rich repeat and pyrin domain-containing protein 3
ASC: the adaptor protein apoptosis-associated speck-like protein containing a CARD (caspase-recruitment domain).
