# Supplementary material for: p31-43 Gliadin Peptide Forms Oligomers and Induces NLRP3 Inflammasome/Caspase 1- Dependent Mucosal Damage in Small Intestine
Source: Front Immunol. 2019 Jan 30;10:31. doi: 10.3389/fimmu.2019.00031 (PMC6363691; doi:10.3389/fimmu.2019.00031)
Supplement: Supplementary file 3 [file Data_Sheet_1.pdf]

Supplementary Material

p31-43 gliadin peptide forms oligomers and induces NLRP3 inflammasome/ caspase 1-dependent mucosal damage in small intestine

María Florencia Gómez Castro[1], Emanuel Miculán[1], María Georgina Herrera[2], Carolina Ruera[1], Federico Perez[1], Eduardo Daniel Prieto[3], Exequiel Barrera[4], Sergio Pantano[4], Paula Carasi[1] and Fernando Gabriel Chirdo[1,*]

***Correspondence:** Fernando Gabriel Chirdo - fchirdo@biol.unlp.edu.ar

Supplementary figures

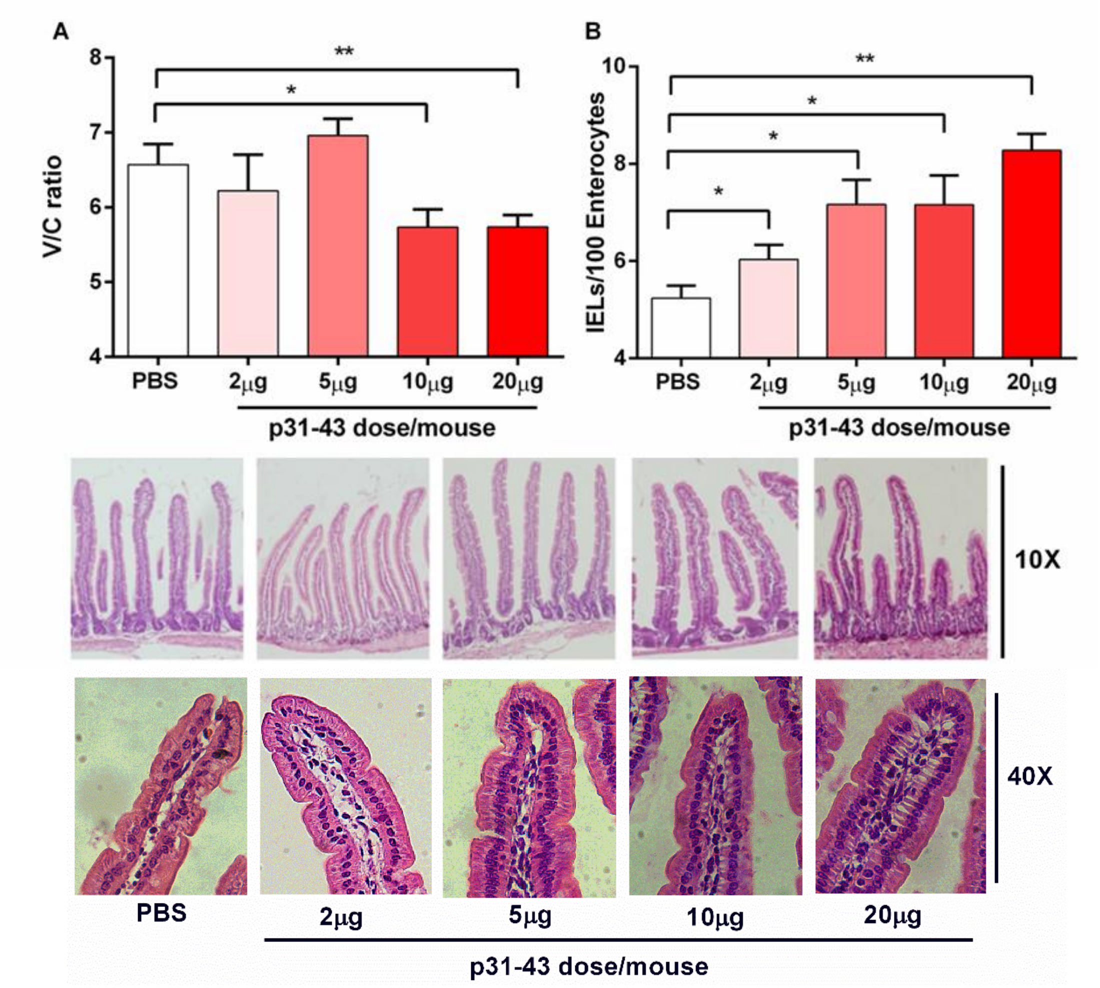

Supplementary Figure 1. Dose dependent induction of enteropathy by feeding p31-43. C57BL/6 mice received 2, 5, 10 or 20µg p31-43 or PBS by oral gavage and enteropathy was assessed 16hr later by counting the number of intraepithelial lymphocytes/100 enterocytes **(A)** and by measuring the ratio between villus length and crypt depth (V/C) **(B)** in H&E stained sections of paraffin-embedded proximal small intestine. Results shown are means ± 1 SEM for 5 mice/group. *p<0.05, ANOVA. **(C)** Representative H&E stained sections.

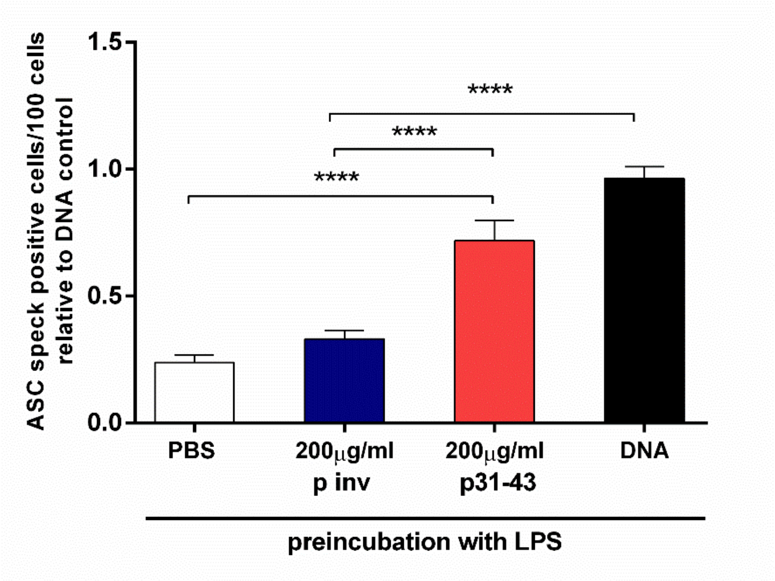

Supplementary Figure 2. ASC speck formation is not induced by inverted peptide. Human THP-1 ASC speck reporter cells were preconditioned for 3h with LPS before addition of 200µg/ml p31-43 or inverted peptide for a further 16h. Positive control cells (DNA) were transfected transiently with bacterial plasmid before addition of LPS. Cells showing ASC specks were assessed by immunofluorescence and expressed as positive cells/100 total cells. Results shown are means ± 1 SEM for three replicates/group. ***p<0.0001, ANOVA.

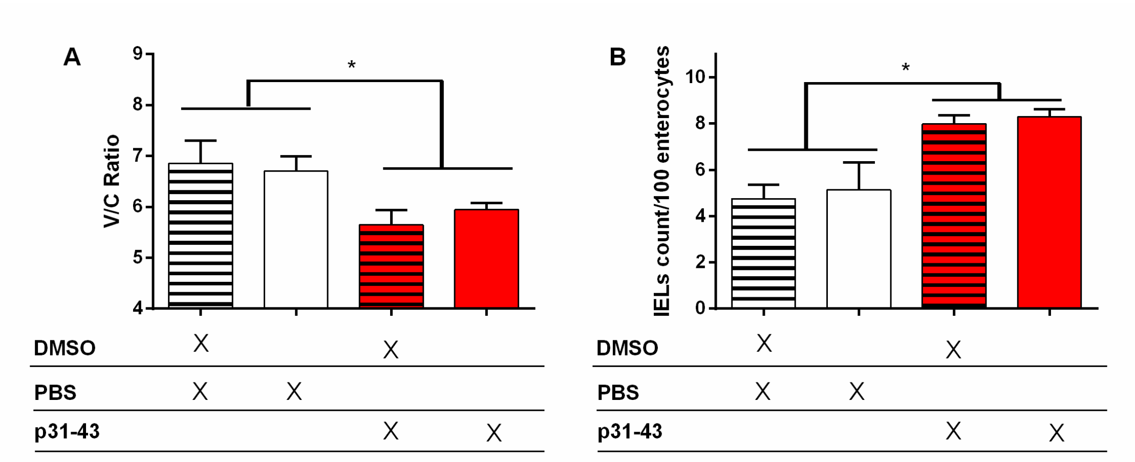

Supplementary Figure 3. Enteropathy induced by p31-43 is not prevented by DMSO. Villus/crypt rations (**A**) and intraepithelial lymphocyte counts (**B**) in the proximal small intestine of C57BL/6 mice fed 20µg p31-43 or PBS that had been treated i.p. 30 minutes earlier with DMSO. Results shown are means ± 1 SEM for 5 mice/group assessed 16hr after feeding p31-43. *p<0.05, ANOVA.
